# Cytosolic SYT/SS18 Isoforms Are Actin-Associated Proteins that Function in Matrix-Specific Adhesion

**DOI:** 10.1371/journal.pone.0006455

**Published:** 2009-07-31

**Authors:** Jaehong Kim, Mei Swee, William C. Parks

**Affiliations:** 1 Center for Lung Biology, University of Washington, Seattle, Washington, United States of America; 2 Division of Biology and Biomedical Sciences, Washington University, St. Louis, Missouri, United States of America; University of Birmingham, United Kingdom

## Abstract

SYT (*SY*novial sarcoma *T*ranslocated gene or SS18) is widely produced as two isoforms, SYT/L and SYT/S, that are thought to function in the nucleus as transcriptional coactivators. Using isoform-specific antibodies, we detected a sizable pool of SYT isoforms in the cytosol where the proteins were organized into filamentous arrays. Actin and actin-associated proteins co-immunoprecipitated with SYT isoforms, which also co-sedimented and co-localized with the actin cytoskeleton in cultured cells and tissues. The association of SYT with actin bundles was extensive yet stopped short of the distal ends at focal adhesions. Disruption of the actin cytoskeleton also led to a breakdown of the filamentous organization of SYT isoforms in the cytosol. RNAi ablation of SYT/L alone or both isoforms markedly impaired formation of stress fibers and focal adhesions but did not affect formation of cortical actin bundles. Furthermore, ablation of SYT led to markedly impaired adhesion and spreading on fibronectin and laminin-111 but not on collagen types I or IV. These findings indicate that cytoplasmic SYT isoforms interact with actin filaments and function in the ability cells to bind and react to specific extracellular matrices.

## Introduction

SYT (*SY*novial sarcoma *T*ranslocated gene or SS18) was discovered as part of a nuclear chimeric protein coded by a t(X;18)(p11.2;q11.2) translocation found in many synovial sarcomas [1]. This translocation fuses the *SYT* gene on chromosome 18 with either the *SSX1*, *SSX2*, or *SSX4* genes on the X chromosome. Findings from several studies have indicated that the SYT/SSX fusion proteins localize to the nucleus and function as potential transcriptional co-activators and effectors of cell proliferation [2]–[10]. Consequently, native SYT, though devoid of a consensus nuclear localization signal, has also been considered to be a nuclear proto-oncogene. Indeed, SYT protein can interact with several transcription factors, such as acute leukemia-associated transcription factor AF10, SWI/SNF proteins, the histone acetyl-transferase p300, and others [2], [11]–[17].

SYT is highly conserved among species [18], [19]. Alternative splicing produces at least four isoforms of SYT, and the most prominent of these are the long (SYT/L; 56 kD) and short (SYT/S; 50.5 kD) isoforms, which differ by inclusion of exon 8 in SYT/L [19]. Although SYT contains potential SH2 and SH3 motifs and an annexin-like QPGY-rich domain, it has no significant homology to other proteins, other than Crest (SS18-like 1), a nuclear protein implicated in neuronal development [20]. SYT is widely expressed during development and in adult tissues [1], [18]. *Syt*-null mice do not develop beyond E9.5 and have profound defects in vascularization, cell migration, neural tube closure, and fusion within the embryonic-maternal membranes [21], [22].

Our interests in SYT sprang from our studies on cell-matrix interactions in tissue repair [23]–[25]. Eid *et al*. [15] reported data suggesting that activation of β_1_ integrins is associated with formation of SYT/p300 complexes. Our findings also demonstrate a role for SYT isoforms in cell-matrix interactions but indicate that cytosolic SYT - the biology of which has not been explored - is the critical pool that functions in adhesion. We found that cytosolic SYT isoforms bind F-actin and are involved in cytoskeletal organization. Ablation of SYT led to impaired assembly of actin stress fibers and cell spreading and adhesion on specific extracellular matrix substrata (fibronectin and laminin-111) but not others (types I and IV collagen). These findings indicate that cytosolic SYT functions in the differential signaling controlling how a cell responds and distinguishes among specific extracellular cues.

## Results

### SYT Antibodies

**Figure 1:**
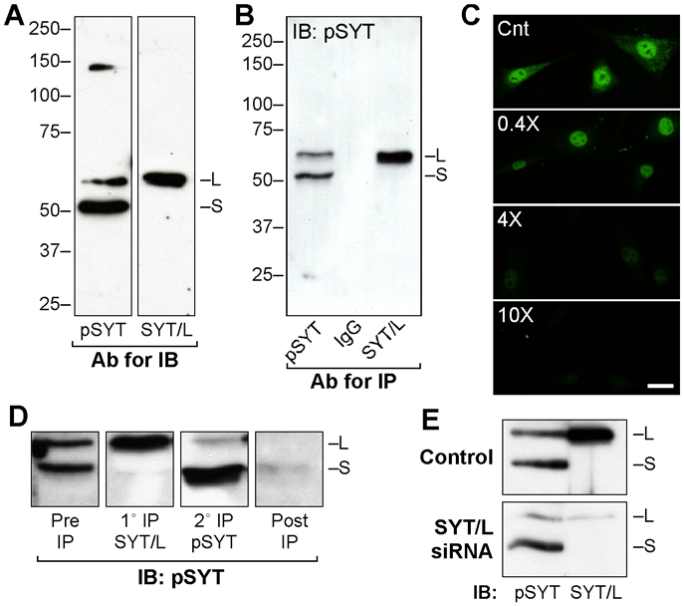
SYT antibodies. (A) Total lysate (40 µg protein) from U2OS cells was immunoblotted (IB) with affinity-purified panSYT antibody (pSYT) or antibody specific for the SYT/L isoform. The pSYT antibody reacted with both SYT/S (S; 50.5 kD) and SYT/L (L; 56 kD) isoforms, whereas the SYT/L antibody reacted only with the long isoform. (B) Total cell lysates were immunoprecipitated with pSYT or SYT/L-specific antibodies or purified IgG. (C) Cos-1 cells were processed for immunofluorescence staining with SYT/L antibody in the presence of increasing molar ratios (relative to antibody) of antigenic peptide. Bar = 20 µm. (D) Total U2OS lysate was immunoprecipitated with SYT/L-specific antibody (1° IP), and the supernatant was re-immunoprecipitated with pSYT (2° IP). Both immunoprecipitates, as well as the starting lysate (Pre IP) and the supernatant after the second immunoprecipitation (Post IP), were immunoblotted with pSYT antibody. (E) Lysates from control U2OS cells or from cells 3 days post transfection with RNAi duplex 894, which targets only SYT/L transcripts (see Figure S1), were immunoblotted with pSYT and SYT/L-specific antibodies.

We generated two polyclonal antibodies: one against the N-terminal half of SYT, which is common to all isoforms, and the other against the peptide sequence coded by exon 8, which is present only in SYT/L. Both antibodies were affinity purified using the recombinant or peptide antigens, and we confirmed the specificity of these antibodies by several approaches. Immunoblotting and immunoprecipitation of human osteosarcoma U2OS cell lysates demonstrated that the panSYT (pSYT) antibody reacted with both SYT/S (50.5 kD) and SYT/L (56 kD) and that the SYT/L-specific antibody detected only SYT/L (Figure 1A, B). Immunofluorescence revealed signal in both the nucleus and cytosol, which was ablated with excess antigen (Figure 1C). Immunoprecipitation with SYT/L-specific antibody cleared lysates of the larger isoform yet retained signal for SYT/S, which was brought down by subsequent immunoprecipitation with pSYT (Figure 1D). After immunodepletion with both antibodies, lysates were cleared of all SYT immunoreactivity.

Targeted RNAi knock-down of the SYT/L isoform (Figure S1) markedly reduced signal for SYT/L detected by either antibody without affecting the levels of SYT/S protein and mRNA (Figure 1E). RNAi knock-down of SYT/S and SYT/L mRNAs markedly reduced the immunofluorescence signal for SYT isoforms detected with the pSYT antibody (Figure S1). Similar results with these assays were obtained with all other cell types examined, including rat lung fibroblasts (RLF), monkey kidney fibroblasts (Cos-1), and mouse fibroblasts (3T3). These data demonstrate that our antibodies specifically recognize SYT isoforms across mammalian species. In addition, even though the epitope of the pSYT antibody is common to all putative isoforms, we did not detect the other, less-abundant SYT isoforms. Indeed, amplification of SYT cDNA confirmed that SYT/S and SYT/L accounted for essentially all mRNAs detected, with only a minor level (<1%) of transcript for the SYT/vs1 isoform (not shown), which lacks exons 7 and 8.

### SYT Isoforms Localize to the Cytoplasm

**Figure 2:**
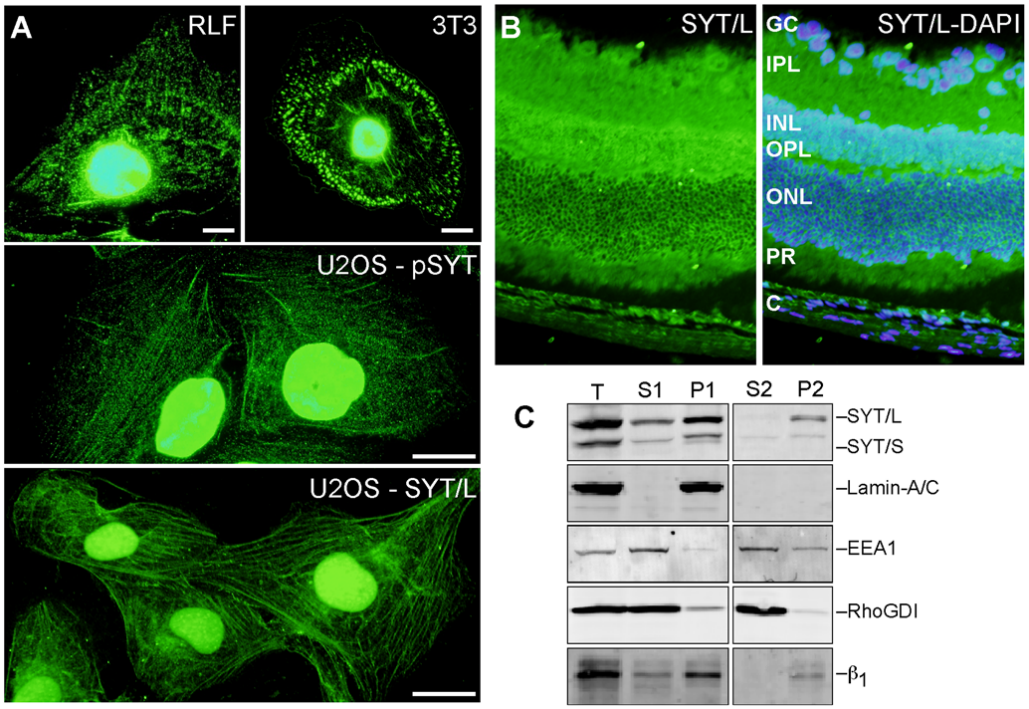
Cytosolic SYT. (A) Rat lung fibroblasts (RFL) and 3T3 fibroblasts were stained with pSYT antibody, and U2OS cells were stained with pSYT or SYT/L-specific antibodies. Bar = 10 µm. (B) Mouse retina was stained for SYT/L. Nuclear signal was seen in ganglion cells (GC) and cells of the inner (INL) and outer (ONL) nuclear layers and within the choroid (C). Prominent cytoplasmic signal was seen within the inner (IPL) and outer (OPL) plexiform layers, the photoreceptors (PR), and the choroid. (C) Total lysate (T) of U2OS cells was separated by centrifugation into cytoplasmic supernatant (S1) and nuclear pellet (P1). The S1 cytosolic fraction was separated further by ultracentrifugation, yielding supernatant (S2) and pellet (P2). Samples were resolved by electrophoresis and immunoblotted with antibodies against total SYT (pSYT), lamin-A/C, EEA-1, RhoGDI, and the β_1_ integrin subunit.

Immunofluorescence with either SYT antibody verified nuclear signal in both cells and tissue (Figure 2A,B); however, signal for SYT protein was also seen in the cytosol of all cells examined, typically in a filamentous pattern (Figure 2A). In addition to U2OS, RLF, and 3T3 cells, we detected filamentous cytosolic SYT in human primary keratinocytes, human HaCaT keratinocytes, human diploid fibroblasts (HDF), human primary fibroblasts (IMR90), human cervical carcinoma cells (HeLa), and monkey kidney fibroblasts (CV-1 and Cos-1) (not shown). Furthermore, prominent signal for cytosolic SYT was detected in all adult mouse tissues examined, including all cell layers of the retina (Figure 2B), intestine, lung, skin, cardiac muscle, spleen, brain, uterus, and testis, among others (not shown). Thus, the cytoplasmic distribution of SYT was seen across tissues, cell types, and species.

Based on cell fractionation, about 20% of total SYT was recovered in the cytosolic fraction (S1 in Figure 2C), which was consistently seen among cell types. Immunoblotting for the nuclear envelope proteins, lamin-A/C, demonstrated no detectable contamination of nuclear contents in the cytosolic fraction. The relatively strong signal for the β_1_ integrin subunit, used as a plasma membrane maker, in the P1 pellet likely indicates that most of the plasma membrane sedimented in nuclear pellet. Indeed, fractionation of biotinylated surface proteins demonstrated that plasma membrane are brought down in nuclear pellet (data not shown).

**Figure 3:**
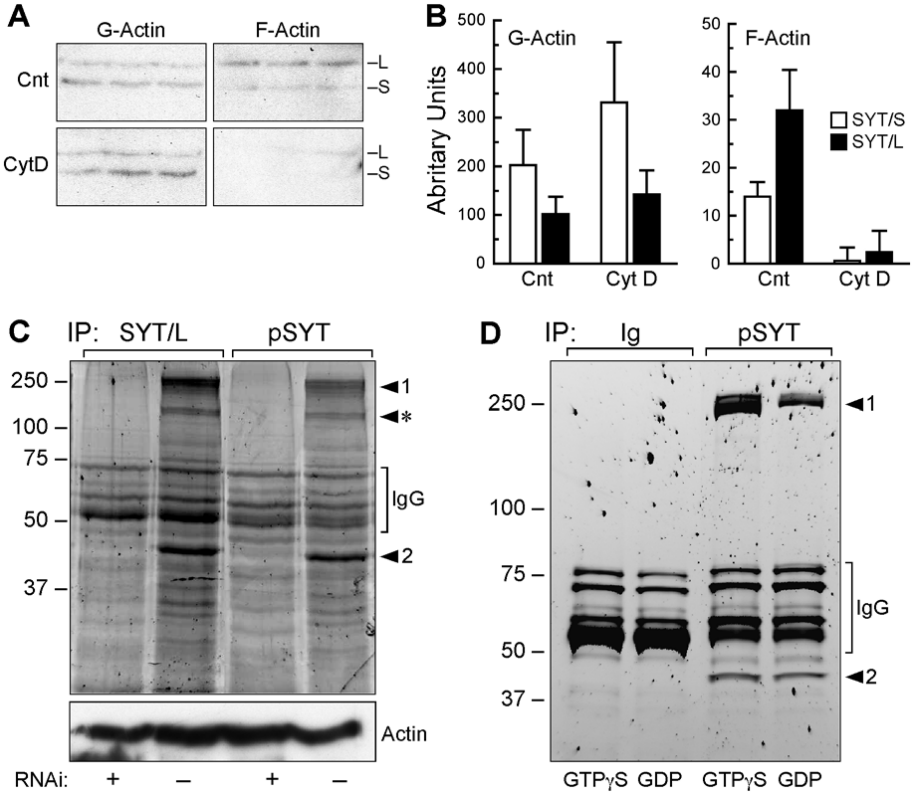
Cytosolic SYT isoforms interact with actin. (A) U2OS cells were treated with vehicle (Cnt) or 4 µM cytochalasin D (Cyt D)for 30 min and then G and F actin pools were isolated and immunoblotted with pSYT antibody. Shown are independent triplicates. (B) Densitometric values of the bands in the gels in panel A were averaged and normalized to the original lysate volume. The data shown are the mean±SEM of the normalized densities from 3 independent experiments. (C) U2OS cells were transfected with panSYT_229_ RNAi duplex (see Figure S1) or control RNAi duplex, and 3 days later, post-nuclear lysates were immunoprecipitated with SYT/L or pSYT antibodies. Lysates were immunoblotted for actin, which demonstrated equal loading among lanes and no decrease in total actin levels in RNAi-ablated cells. (D) Cos-1 lysates were pre-incubated with GTP-γS or GDP for 30 min at 30°C before immunoprecipitation with control IgG or pSYT antibody. Co-immunoprecipitated bands in both gels were visualized by SYPRO Ruby staining, and the specific bands (1, 2, *) were excised, sequenced by MS/MS (see Figure S2), and identified as myosin heavy chains 9 and 10 (1), actin (2), and hnRNP U (*). The nonspecific bands migrating between 50–80 kD are IgG subunits.

Further fractionation of the cytosolic sample (S1) by ultracentrifugation resulted in essentially all cytosolic SYT in the pellet (P2), which was enriched with cytoskeletal and the remaining membrane components (β_1_ integrin subunit). No signal for SYT isoforms was detected in the ultra high-speed supernatant (S2), which contained soluble proteins (e.g., RhoGDI) and vesicles (EEA1). Because we did not detect SYT protein on plasma membrane by immunofluorescence (Figure 2A), we hypothesized that cytosolic SYT isoforms were associated with cytoskeletal components in the P2 pellet. Consistent with this idea, exposure to cytochalasin D, which depolymerizes filamentous actin, abolished the cosedimentation of SYT isoforms with the cytoskeleton (Figure 3A,B). SYT isoforms sedimented with filamentous (F) actin pools; however, in the presence of cytochalasin D, the level of SYT in the F-actin fraction was markedly reduced. These findings indicate that cytosolic SYT isoforms localized in a subcellular compartment associated with actin cytoskeleton and not with a membrane compartment.

### Cytosolic SYT is an Actin-associated Protein

We immunoprecipitated SYT from cytosolic lysates and identified co-precipitated proteins by tandem mass spectrometry (MS/MS). Immunoprecipitation of U2OS or Cos-1 lysates with pSYT or SYT/L-specific antibodies consistently brought down two prominent specific bands (Figure 3C,D). The larger of these resolved as a doublet (Band 1) and contained myosin II heavy chains (MHC) 9 and 10 and the faster migrating band (Band 2) was identified as actin. MS/MS sequencing of the excised bands revealed several peptides (9 to 19) for these proteins (Figure S2), providing strong evidence for their identity. Importantly, no other peptides were detected in the excised bands. To assess the specificity of these interactions, we compared immunoprecipitates of control cells and cells with RNAi-ablation of SYT isoforms (verification and controls for the RNAi's are discussed below). The total level of actin was not affected by SYT ablation, but actin and MHCs were not brought down with SYT antibodies from lysates of RNAi knock-down cells (Figure 3C). In some co-immunoprecipitates, we identified hnRNP U (Band *), a ribonucleoprotein that interacts with actin [26]. MHC 9 and 10 and actin also co-immunoprecipitated with SYT from lysates of 3T3, Cos-1, RLF, and HeLa cells (data not shown).

Rho GTPase controls the interaction between actin and myosin II [27]–[29]. To assess if the interaction of SYT isoforms with actin and MHCs was dependent on GTPase activity, we incubated lysates with nonhydrolyzable GTP-γS or excess GDP to increase or decrease GTPase activity, respectively, and then immunoprecipitated with pSYT antibody. Although many interactions may be disturbed by GTP-γS or GDP, the recovery of co-immunoprecipitated MHC 9 and 10 was 4.2-fold greater from lysates containing GTP-γS than from lysates with GDP (Figure 3D). In contrast, the level of co-immunoprecipitated actin differed less than 5% between conditions and among experiments (Figure 3D). These co-immunoprecipitation results, along with the cosedimentation with F-actin, were consistently seen among experiments and indicate that SYT isoforms interact, directly or indirectly, with actin and that actin-bound MHCs are brought down in a higher order complex.

### SYT Colocalizes with F-actin

**Figure 4:**
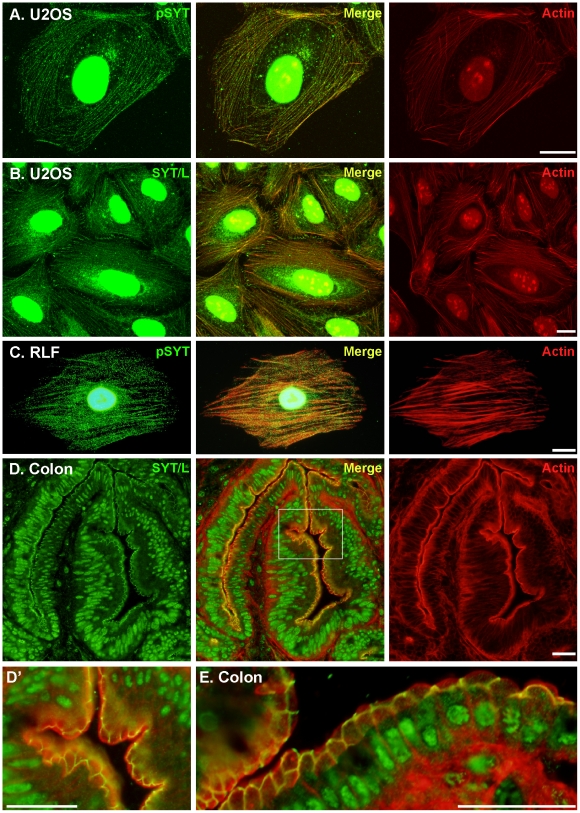
Cytosolic SYT colocalizes with actin filaments. U2OS (A,B) and RLF (C) cells and mouse colon (D,E) were stained with rhodamine-conjugated phalloidin (actin) and pSYT or SYT/L-specific antibody. (A-C) Signal for SYT proteins colocalized extensively with the well formed arrays of stress fibers in all cells. (D) SYT also colocalized with the actin network at the apical edge of colon epithelial cells. The boxed area in the merged micrograph is expanded in panel D'. (E) A section of colon mucosa form another mouse showing nearly complete overlap with filamentous actin at the apical-lateral borders of epithelia cells. Bars = 10 µm for panels A-C and 25 µm for D and E.

**Figure 5:**
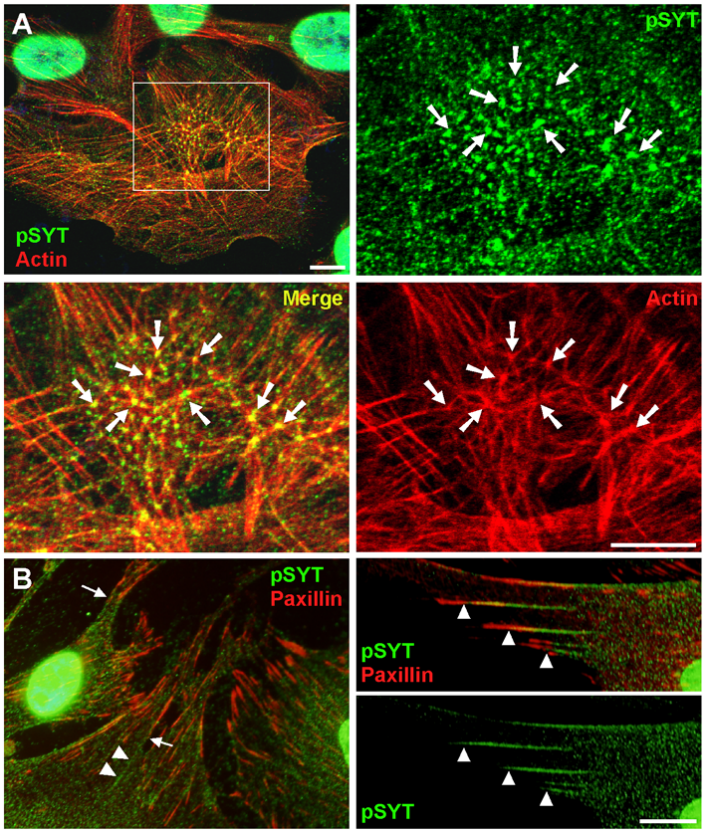
SYT accumulates at junctions of actin filaments. (A) U2OS cells were stained with rhodamine-conjugated phalloidin (red) and with pSYT antibody (green) and viewed by confocal microscopy. The micrographs shown are a single 30-nm z-section through the center of a cell. The boxed area in the merged micrograph (upper left panel) is shown under high magnification in the other three. Arrows point to bends and junctions in actin filaments with strong co-localization of SYT. (B) U2OS cells were immunostained with pSYT (green) and paxillin (red) antibodies. Arrowheads demarcate the junctions between paxillin-positive focal adhesions and SYT staining.

Cytosolic SYT was organized into filamentous strands, which were seen in all cell types examined (Figure 4A-C). Consistent with the co-immunoprecipitation and co-sedimentation data (Figure 3), we saw extensive colocalization of SYT with filamentous actin (Figure 4), especially at branch points (Figure 5A). Colocalization of SYT with F-actin was seen in every cell type examined and was revealed with either pSYT or SYT/L-specific antibodies (Figure 4, 5A). The association of SYT strands with actin filaments diminished gradually toward the distal ends of stress fibers at the cell periphery, and as demonstrated by a lack of merged signal with paxillin, SYT did not extend into focal adhesions (Figure 5B). Colocalization of SYT with the actin cytoskeleton was also seen in many tissues and was particularly evident at the apical-lateral border edge of the intestinal epithelium (Figure 4D,E), uterus, seminiferous tubules, kidney tubules, and more (not shown).

**Figure 6:**
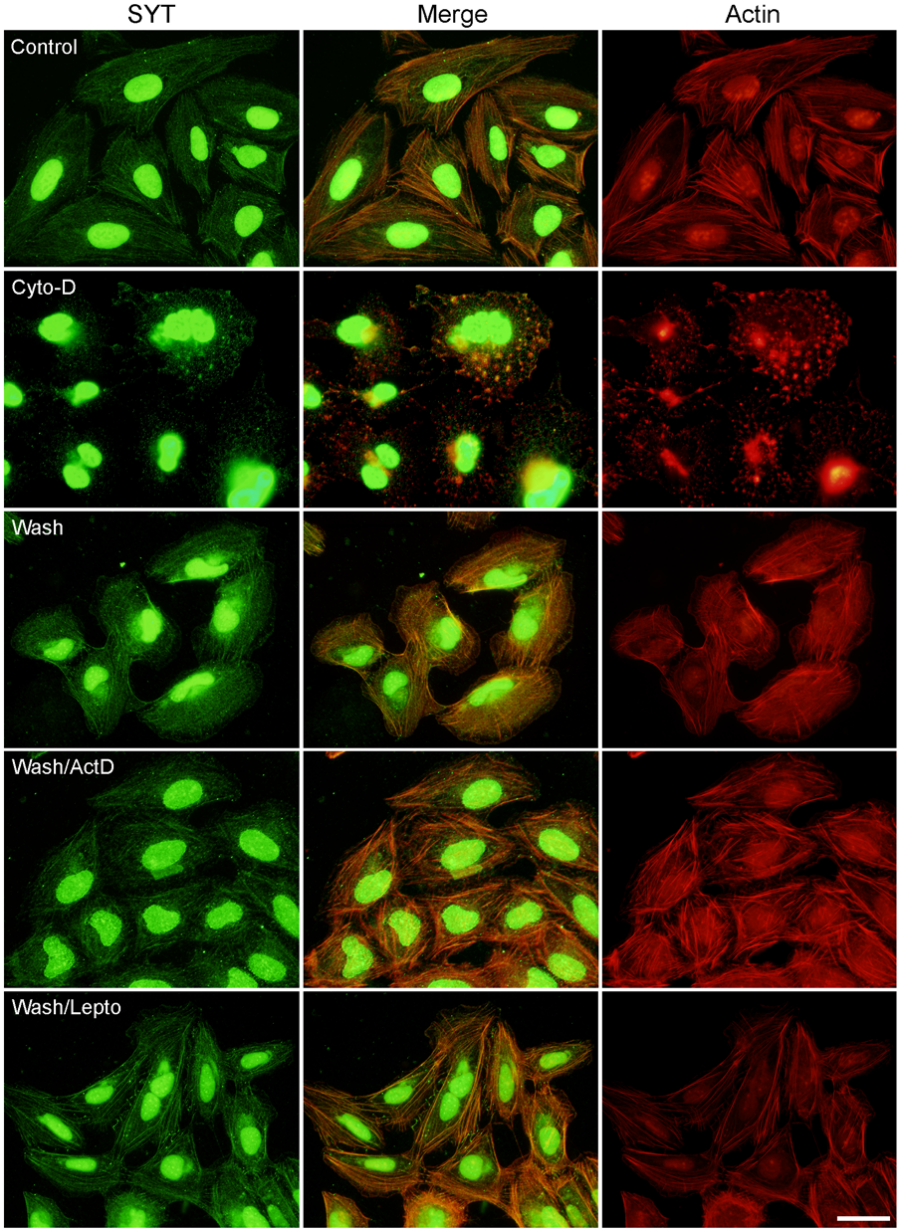
Organization of cytosolic SYT is dependent on actin polymerization. U2OS cells were treated with DMSO (Control) or 4 µM cytochalasin D (Cyto-D) for 90 min, washed for 1 h in complete medium (Wash), and stained with pSYT antibody (green) and rhodamine-conjugated phalloidin (red). Other cultures were pretreated with 1.25 µg/ml actinomycin D or with 20 µg/ml leptomycin B for 5 h before addition of cytochalasin D; the presence of these compounds was maintained during the cytochalasin D treatment and subsequent wash steps. Bar = 15 µm.

We assess if SYT was linked to actin polymerization. Both SYT strands and F-actin collapsed upon exposure to cytochalasin D (Figure 6) or latrunculin (data not shown). After a 1-h wash to remove the depolymerization agents, SYT strand organization recovered concomitantly with the reestablishment of an actin cytoskeleton. Reformation of SYT strands and their colocalization with actin filaments was not disrupted by actinomycin D, an RNA polymerase inhibitor, or by leptomycin B, an antagonist of Crm1-mediated nuclear export, added before, with, and after cytochalasin D (Figure 6). These data indicate that active transcription and nuclear export are not required for formation of cytosolic SYT strands or their association with the actin cytoskeleton. Furthermore, SYT strands did not colocalize with tubulin, and their structure remained intact after disruption of microtubules (Figure S3).

### SYT Functions in Assembly of F-Actin

We used RNAi knock-down to assess if SYT isoforms function in actin assembly and cell adhesion. We first determined the turnover rate of SYT isoforms by pulse-chasing U2OS, Cos-1, and HeLa cells with [^35^S]methionine/cysteine. The half-life of SYT/L was 14.5 h and 13.6 h for SYT/S, and these rates did not vary appreciably among cell types (data not shown). We designed seven RNAi duplexes targeting sequences common to both SYT/L and SYT/S (panSYT) mRNAs and two RNAi duplexes specific for SYT/L mRNA (Figure S1). Of the panSYT duplexes, RNAi 97, 229, 471, and 503 reproducibly reduced total SYT mRNA levels>95% and total SYT protein to levels undetectable by immunoblotting at 3 days post-transfection (>5 half-lives) of U2OS and Cos-1 cells (Figure S1). A few cells transfected with panSYT_229_ had weak residual nuclear staining for SYT but no cytosolic signal. The immunofluorescence data also demonstrated that the transfection efficiency of the RNAi duplexes was essentially 100%. We obtained partial (50–90%) knock-down of total SYT with RNAi duplexes 180, 414, and 838. Specific ablation of SYT/L mRNA and protein was achieved with the SYT/L-specific RNAi duplexes 894 and 933, which did not affect expression of the SYT/S isoform (Figure 1E; Figure S1). As shown below, we obtained the same phenotype with all effective RNAi duplexes.

**Figure 7:**
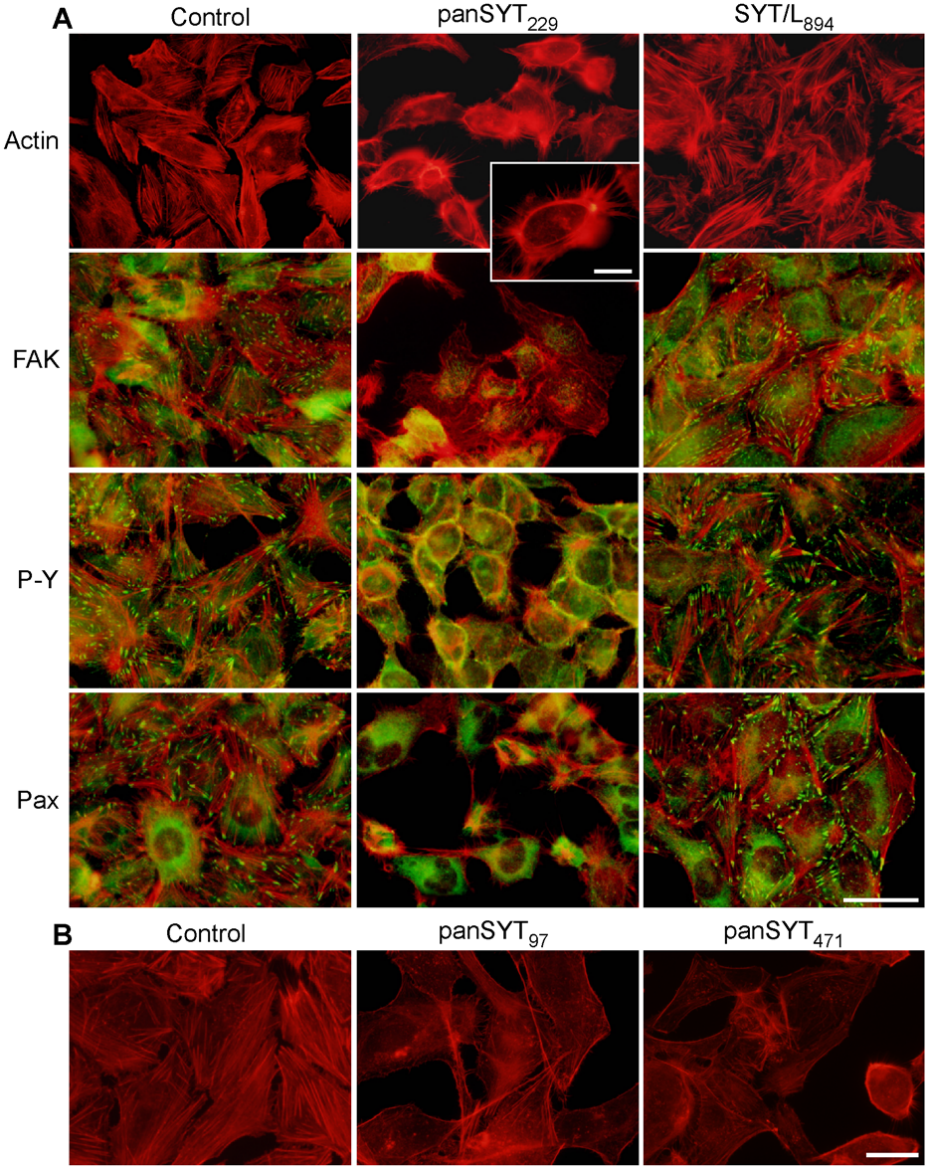
Ablation of SYTs impairs formation of stress fibers and focal adhesions. (A) At 3 d post transfection with control, panSYT_229_, or SYT/L_894_-specific RNAi duplexes, U2OS cells were stained with phalloidin (actin; red) or co-stained with phalloidin and FAK, pan-phosphotyrosine, or paxillin antibodies (green). The inset in the middle column highlights the exaggerated filopodia, presence of cortical F-actin, and absence of stress fibers in cells transfected with panSYT_229_ RNAi duplexes. (B) Similar experiment with different (panSYT_97_ and panSYT_471_) panSYT RNAI duplexes. Bars = 20 µm or 5 µm (inset in A).

We transfected U2OS cells with control (similar GC content), panSYT_97_, panSYT_229_, panSYT_471_, or SYT/L-specific RNAi duplex 894 and assessed the formation of stress fibers 3 days later (Figure 7). Whereas well-formed cytoskeleton was seen in cells transfected with control or SYT/L-specific RNAi's, stress fibers were not detected in cells transfected with panSYT RNAi's (Figure 7). Total actin levels were not affected in RNAi-ablated cells (Figure 3A). Furthermore, in the absence of total SYT, cortical actin was still detected and we noticed robust membrane ruffling and exaggerated filopodia formation (Figure 7, inset), indicating that SYT is not needed for actin polymerization within certain compartments. Importantly, a similar phenotype (lack of stress fibers but retention of cortical actin bundles) was recently reported for fibroblasts isolated from *Syt*-null mouse embryos [22]. In addition, focal adhesions, identified by staining for FAK, paxillin, and phosphotyrosine, were disrupted in panSYT RNAi-transfected cells (Figure 7A). We saw no overt change in stress fiber formation or focal adhesion markers in cells with specific knock-down of SYT/L, suggesting that SYT/S compensates for loss of the larger isoform in adherent cells.

### Cytoplasmic vs. Nuclear SYT

**Figure 8:**
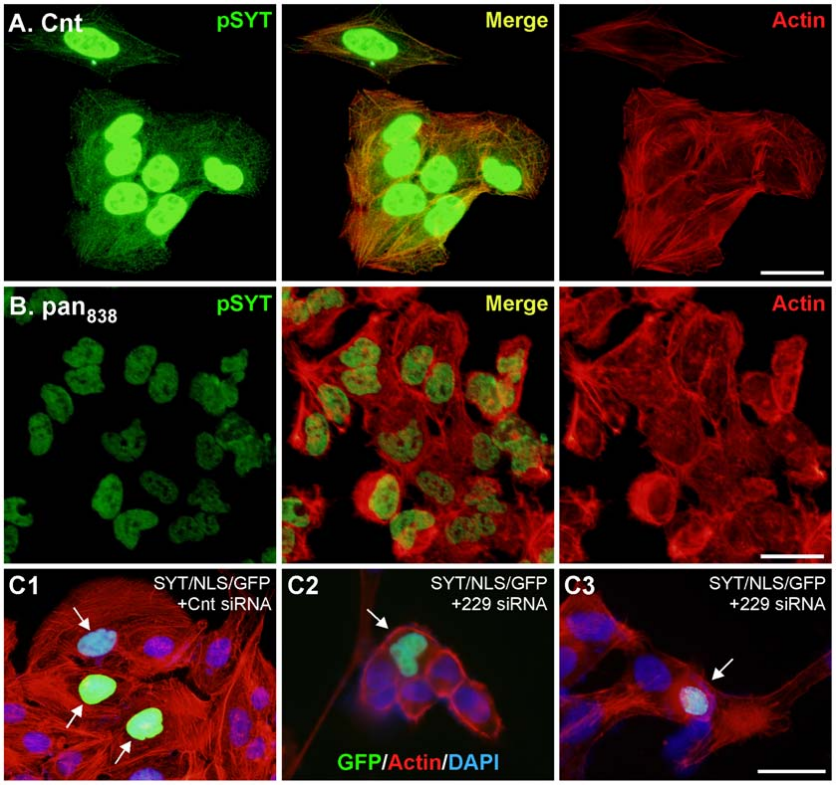
Nuclear SYT is not required for stress fiber formation. U2OS cells were transfected with control (A) or panSYT_838_ (B) RNAi duplexes and stained 72 h later with phalloidin (red) and pSYT antibody (green). Despite persistent levels of nuclear SYT in panSYT_838_ knock-down cells, stress fiber formation was not recovered. (C) U2OS cells were transfected with pCMV/GFP/SYT/NLS and 10 h later with control (Cnt) or panSYT_229_ RNAi duplexes and stained 72 h later with phalloidin (red) and DAPI (blue). Because the plasmid transfection is less efficient than that for RNAi duplexes, GFP fluorescence was seen in only a subset of cells (arrows). Bars = 20 µm.

Findings from three studies indicated that stress fiber formation required cytosolic SYT and was not dependent on the nuclear stores. First, as stated above, transfection with RNAi panSYT_838_ resulted in partial knock down of total SYT levels, and as demonstrated by immunofluorescence, the residual SYT was detected only in the nucleus (Figure 8B). Despite the presence of nuclear SYT, stress fiber formation was disrupted in these cells as thoroughly as seen in cells transfected with the more effective RNAi duplexes.

Second, we expressed full-length SYT/S with a triple repeat of the SV40 large T antigen nuclear localization sequence (NLS) and a GFP tag linked to the N-terminus. We transfected this chimera (SYT/NLS/GFP) or control (NLS/GFP) plasmid into U2OS cells, and 10 h later, transfected with panSYT_229_ or control RNAi duplexes. In cells with control RNAi, we detected GFP signal only in the nucleus of a subset of cells (Figure 8C1). In panSYT_229_-transfected cells, we also detected GFP only in the nucleus, indicating that the over-expressed recombinant transcript overwhelmed RNAi knock-down, but we saw no evidence of stress fiber recovery in these cells (Figure 8C2,3).

Third, in the presence of actinomycin D and leptomycin B, colocalization of SYT with stress fibers was restored after removal of cytochalasin D (Figure 6). These findings indicate that nuclear events like transcription and export of nuclear proteins do not affect the colocalization of SYT with stress fibers. A common conclusion from these three studies was that cytosolic SYT isoforms regulate actin organization independently of the nuclear pools of this protein.

### SYT Controls Matrix-specific Adhesion

Our findings that SYT was needed for stress fiber and focal adhesion formation suggested a role in cell-matrix adhesion. At 1 and 2 days post-transfection with RNAi duplexes, the total number of cells did not differ among cells transfected with control, panSYT_229_, or SYT/L-specific RNAi duplexes. However, between 2 and 3-days post-transfection, the number of control cells had nearly doubled, whereas we saw a smaller increase in the total number of cells transfected with panSYT_229_ or SYT/L-specific RNAi (data not shown). Because we detected no difference in the apoptotic or proliferative indices of transfected cells (data not shown), we conclude that the reduction in the number of cells on the surface at day 3 was a consequence of impaired adhesion.

**Figure 9:**
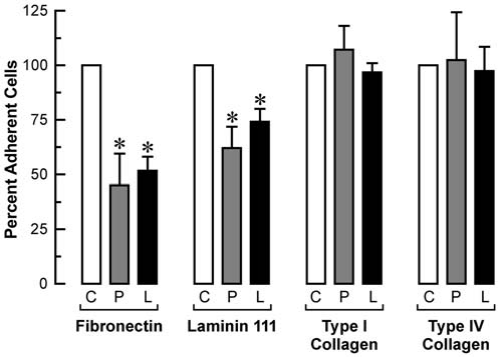
Ablation of SYT inhibits adhesion on specific extracellular matrices. At 3 d post transfection, U2OS cells were harvested, and equal number (2, 3, or 3.15×10^5^ per experiment) of viable cells were plated on 6-well plates precoated with fibronectin, laminin-111, type I collagen, or type IV collagen. Adherent cells were detached 90 min later and counted. For control cells, the absolute percent adhesion (mean±SEM) across experiments was 68.1%±3.5 on fibronectin; 58.1%±4.6 on laminin-111; 51.7%±3.0 on type I collagen; and 47.0%±1.2 on type IV collagen. Each datum point was normalized to the averaged percent of control cells (control RNAi). Then all data points were adjusted to their matrix-specific controls set at 100%. Both graphs show the mean±SEM of independent experiments (*n* = 3), and each datum point is the average of triplicate determinations per condition per experiment. *p<0.05 relative to scrambled RNAi transfected cells.

**Figure 10:**
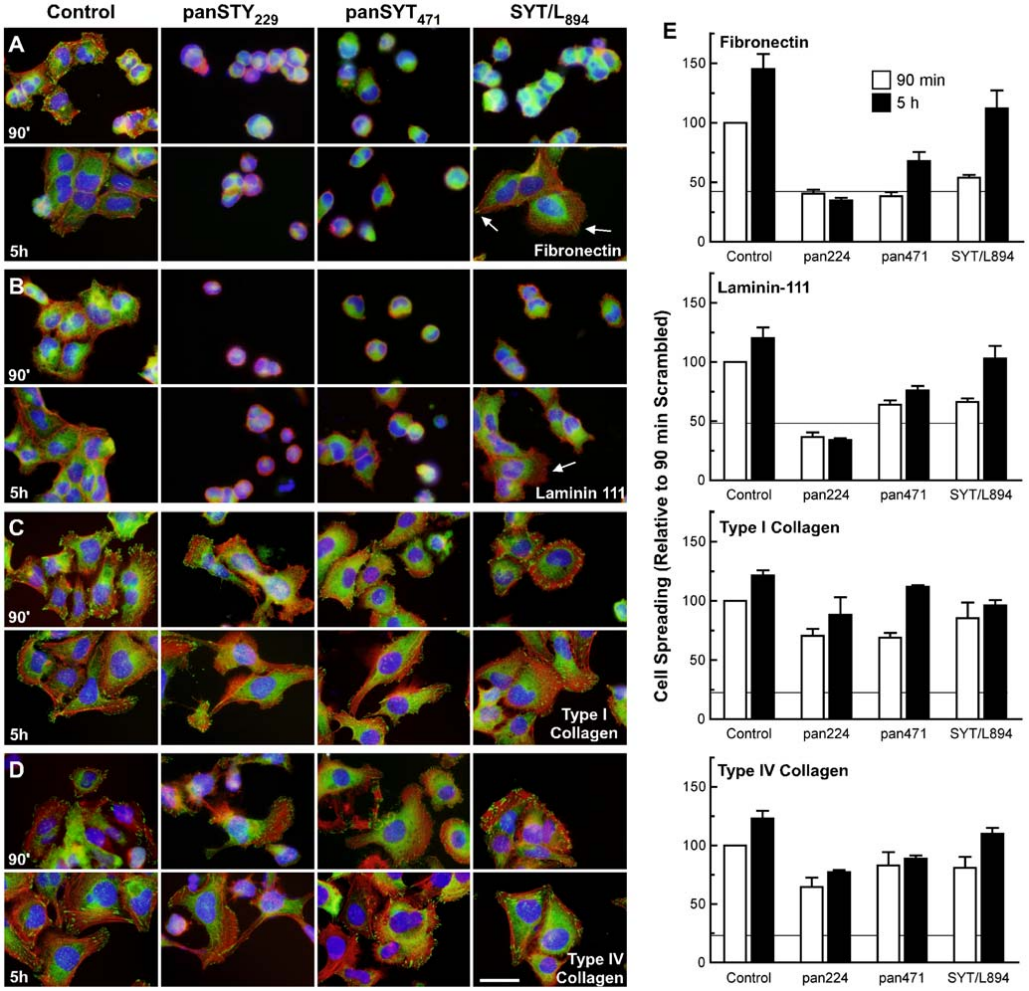
SYT is required for cell spreading on fibronectin and laminin-111 but not on collagen. U2OS cells were transfected with control, panSYT_229_, panSYT_471_, or SYT/L_894_ RNAi duplexes. Three days later, the transfected cells were harvested and replated on chamber slides precoated with (A) fibronectin, (B) laminin-111, (C) type I collagen, or (D) type IV collagen. After a 90-min or 5-h incubation, the slides were gently washed with PBS and stained with rhodamine-conjugated phalloidin (red), DAPI (blue) and anti-paxillin antibody (green). In some SYT/L RNAi-transfected cells, cell spreading and few focal adhesions (arrows) were seen at 5 h post-plating. Bar = 20 µm. (E) Cell spreading was quantified by measuring and averaging the total cell area at the given times on the various substrata. For each condition, cells within 10 randomly selected areas (×20) from each of 3 experiments (>300 cells per point) were measured. Data are the mean±SEM and are normalized to the value of the 90-min controls for each matrix. The horizontal line in each graph represents the extent of cell spreading at 15 min post-plating on the various matrices.

We then assessed if SYT was required for the ability of cells to adhere and spread on specific extracellular matrix substrata. Three days post-transfection, adherent U2OS cells were harvested, and an equal number of viable cells were replated on matrix-coated wells. Cells with ablation of total SYT (panSYT_229_ or panSYT_471_) or SYT/L only (SYT/L_894_) had impaired adhesion to fibronectin and laminin-111, but we saw no decrease in adhesion of cells plated on type I or type IV collagens (Figure 9). Cells with ablation of total SYT (panSYT_229_, panSYT_471_) and SYT/L only (SYT/L_894_) remained rounded and did not spread on fibronectin or on laminin-111 but did on the collagen matrices (Figure 10; Figure S4, S5). Co-staining with phalloidin and paxillin antibody demonstrated that stress fibers and focal adhesions were markedly underdeveloped in all knocked-down cells on fibronectin or laminin-111 but were well formed in cells plated on type I or IV collagen (Figure 10; Figures S4, S5). These results indicate that SYT/S was not able to compensate for the lack of SYT/L within 90 min after replating. At 5 h post-plating, cells with selective ablation of the SYT/L isoform did spread similar to control cells but with only occasional evidence of focal adhesion formation (Figure 10). In knock-down cells on collagen, immunostaining for phosphotyrosine was localized to adhesion complexes but was diffuse in cells on fibronectin (Figure S5). These findings demonstrate that SYT functions in the cytoskeletal machinery used to differentiate among extracellular signals.

In knock-down cells on collagen, immunostaining for phosphotyrosine was localized adhesion complexes but was diffuse in cells on fibronectin (Figure S5), suggesting a disturbance in downstream pathways of fibronectin-mediated cell-matrix adhesion. Ablation of cytosolic SYTs in U2OS cells cotransfected with panSYT_229_ RNAi duplex and GFP/SYT/NLS plasmid or transfected with panSYT_838_ showed reduced spreading in addition to a defect in stress fiber formation (Figure 8B,C). These findings indicate that cytosolic SYT functions in the cytoskeletal machinery used to differentiate among extracellular signals for cell-matrix adhesions.

## Discussion

We report that SYT isoforms are present in the cytosol and interact with F-actin, particularly with stress fibers in a variety of cells and tissues. Our data indicate that cytosolic SYT functions to modulate cytoskeletal organization and adhesion in response to specific matrix substrata. SYT was prominently associated with stress fibers in all tested cells and especially in U2OS cells that have distinct arrays of stress fibers, inclusive of ventral, dorsal, and transverse bundles of actin filaments [30]. The association of SYT diminished near the cell periphery and stopped short of focal adhesions.

The interactions between SYT and actin stress fibers were reciprocal though not completely equal. As demonstrated in our studies with cytochalasin D and latrunculin, actin polymerization was required for formation of SYT strands. However, although knock-down of SYT blocked stress fiber formation, it did not impact total actin polymerization as gauged by exaggerated filopodia and cortical actin filaments, a phenotype also seen in *Syt*-null fibroblasts [22]. Furthermore, we easily detected actin by Sypro Ruby staining in immunoprecipitations with SYT antibodies (Figure 3A, B), but we did not see the SYT proteins themselves. Although the isoforms could have been masked by the IgG heavy chains (a seemingly parsimonious explanation), the lack of visible stained bands at 50 and 55 kD in our immunoprecipitates suggests that the stoichiometry of the SYT:actin interaction favors actin.

In polarized cells *in vivo*, such as like intestinal epithelial cells, SYT colocalized with circumferential actin belt prominently running along apical-lateral edges (Figure 4D,E), and based on RNAi-cell culture studies, SYT may have an important role in the organization and function of these cytoskeletal elements. In support of this idea, contraction of circumferential actin belt in lining epithelial cells is critical for closure of the neural tube [31], and *Syt*-null mouse embryos have a marked defect in neural tube closure (Kimura, 2009). Thus, it is possible that this phenotype arises due to lack of an association of SYT with filamentous actin.

Our data also demonstrate that cytosolic SYT functions in controlling adhesion to specific extracellular matrix substrates. Integrins - heterodimers of α and β subunits - are the key receptors that cells use to sense and respond to the pericellular extracellular matrix environment. The ability of integrins to bind matrix ligands and to transduce information of what they have encountered is controlled by upstream (inside-out) receptor activation, formation and maturation of focal complexes and adhesions, and downstream (outside-in) signal transduction, and each of these processes has been functionally linked to the actin cytoskeleton [29], [32]–[34]. We speculate that SYT functions primarily upstream of integrin activation and/or focal adhesion formation to affect the activity of specific cell-matrix interactions. The observations that filopodia and cortical actin networks formed in SYT-ablated cells indicate that many of the mechanisms downstream of integrin ligation - particularly events mediated by the small GTPases Rac and cdc42 [29] - are not dependent on SYT. Furthermore, because focal adhesions and stress fibers formed in SYT-ablated cells on types I and IV collagen but not on fibronectin and laminin-111 and because the integrins that bind fibronectin and laminins are distinct from those that recognize collagens I and IV [34], SYT may function selectively with the fibronectin/laminin-binding integrins. Consistent with these ideas and our findings, Eid *et al*. [15] reported that over-expression of dominant-negative SYT led to decreased activation of β_1_ integrins and adhesion to fibronectin.

The observations that SYT-ablated cells bound to and spread on collagen and formed focal adhesions suggests that activation of collagen-binding integrins is not dependent on SYT and/or that downstream signaling differs between fibronectin- and laminin-binding integrins and collagen integrins. Although ligation of other integrins can either inhibit or promote the ability of α_2_β_1_ to bind collagen and, hence, signal [35]–[37], an upstream role for the actin cytoskeleton has not been assessed for activation of collagen-binding integrins. Integrin downstream signaling involves many proteins, such as FAK, paxillin, α-actinin, vinculin, and many others, that affect actin organization, among other processes [33], [34]. Although a few studies have suggested that different integrins mediate different effects on Rho GTPase activity [38], [39], it is not clear how signaling downstream of one matrix-integrin contact differs from that mediated by another interaction or how multiple simultaneous signals are integrated. Our future studies are aimed at assessing how cytosolic SYT functions to control adhesion to specific matrix proteins.

## Materials and Methods

### Cells and Tissue

Human diploid fibroblasts (HDF) and NIH-3T3, U2OS, Cos-1, IRM-90, HeLa, and 293 cells were purchased from ATCC (Manassas VA). Rat lung fibroblasts (RLF) were isolated as described [40]. Cos-1 and 3T3 cells were grown with DMEM, and U2OS cells, RLF, and HDF were grown in McCoy's 5A (ATCC), F12K (ATCC), and EMEM (Mediatech, Herndon VA) media, respectively. All culture media were supplemented with 10% FBS. In some studies, cells were treated with 3 µg/ml nocodazole (Calbiochem, La Jolla CA), 4 µM cytochalasin D (Calbiochem), 2 µM latrunculin (Calbiochem), 20 µg/ml leptomycin B (EMD, San Diego, CA), or 1.25 µg/ml actinomycin D (EMD). Tissues were harvested from 6-week old C57BL/6 mice and processed for immunostaining as described [41].

### SYT Protein and Antibodies

Full length cDNAs of human SYT/L and SYT/S were cloned by reverse transcription-PCR (RT-PCR) of total RNA from 293 cells using primers shown in Figure S6. RNA was isolated with Trizol (Invitrogen, Carlsbad, CA) and transcribed into cDNA synthesis Superscript II (Invitrogen). SYT cDNAs were amplified with AccuPrime™ taq (Invitrogen) with 28–30 cycles using primers shown in Figure S6. PCR products were resolved through 1.2% agarose, and bands corresponding to SYT/S and SYT/L were eluted and sequenced.

To generate antibodies reactive to both isoforms (panSYT), the 5′ half of SYT cDNA, upstream of the alternatively spliced exon 8, was amplified with the primers (Figure S6) and subcloned into pGEX-KG and transformed into BL-21 competent cells (Stratagene, La Jolla CA). After a 2-h induction with IPTG, GST-tagged fusion proteins were isolated from bacterial lysate by glutathione agarose (Sigma, St. Louis MO) affinity chromatography. The GST tag was removed using a Thrombin Cleavage Capture kit (Novagen, Madison WI) and glutathione agarose batch chromatography and removal was confirmed by immunoblotting with anti-GST (Upstate, Lake Placid NY). To generate an SYT/L-specific antibody, a 15-amino acid peptide (YPEQGYDRPYEDSSQ), representing the sequence coded by exon 8, was selected using the Peptideselect™ design tool (peptideselect.invitrogen.com/peptide/). Purified recombinant SYT (8 mg) and SYT/L peptide (20 mg) were sent to Evoquest™ (Invitrogen) to raise polyclonal antibodies in rabbits and for affinity purification.

### Immunoprecipitation

Cells were grown to about 80% confluency, washed, and dounced homogenized and centrifuged or lysed in MLB buffer (Upstate; 25 mM HEPES, pH 7.5, 150 mM NaCl, 1% Igepal CA-630, 10 mM MgCl_2_, 1 mM EDTA, 2% glycerol, 1 mM ATP). To affect GTPase activity, lysates were adjusted to 10 mM EDTA and 100 µM GTPγS or 1 mM GDP and incubated for 30 min at 30°C. The reactions were stopped by chilling on ice and adding 60 mM MgCl_2_. Primary antibodies (1 µg) were incubated overnight with 600 µg (total protein) of post-nuclear or whole cell lysates in 600 µl. Protein quantitation was done with a BCA assay kit (Pierce, Rockford IL). For co-immunoprecipitation studies, antibody-bound complexes were brought down with Protein G PLUS-Agarose (Santa Cruz, Santa Cruz CA). Immunoprecipitated products were washed 3 times with 1 ml RIPA buffer (50 mM Tris-HCl, pH 7.4, 150 mM NaCl, 0.25% deoxycholate 1% NP-40, 1 mM EDTA) and dissolved in SDS loading buffer at 95°C for 10 min and resolved through SDS-PAGE gels (8, 10, or 12% acrylamide). Co-immunoprecipitated bands were identified by staining with Sypro Ruby (BioRad, Hercules CA), excised and sent to Midwest Bio Services (Overland Park KS) for sequencing by nano LC/MS/MS.

### Immunoblotting

After electrophoresis, gels were equilibrated in TGMS buffer (1X Tris Glycine buffer, 20% methanol, 0.2% SDS), and proteins transferred to PVDF membranes. True blot™ (e-Biosciences, San Diego CA) was used to obscure detection of antibody chains. Gels and filters were processed as described [25]. Band densities were measured with NIH Image J software (rsb.info.nih.gov/ij).

### Immunofluorescence/Confocal

Cells were plated on CC2-treated Lab-Tek™II 4-well chamber slides (Nalge Nunc, Rochester NY), washed with PBS, fixed for 10 min in buffered formalin, and permeabilized in 2% BSA, 0.2% Triton-X 100, PBS. Fixed cells were incubated for 30 min with Image-iT™FX Signal Enhancer (Invitrogen) to minimize background fluorescence and then overnight with primary antibodies at 4°C in a humidified chamber. PanSYT and SYT/L affinity-purified antibodies were used at a final concentration of 0.83 ng/ml. For controls, we used non-immunized purified rabbit IgG or competition with excess antigenic peptide. Antibodies against FAK, paxillin, actin, and tubulin were from BD Pharmagen (San Diego CA) and pan-phosphotyrosine antibody 4G10 was from Upstate (Charlottesville, VA). Bound primary antibodies were detected with Alexa Fluor 488 anti-rabbit or Alexa Fluor 568 anti-mouse antibodies (Molecular Probes, Eugene OR) diluted 1∶1000. Filamentous actin (F-actin) was detected with rhodamine-conjugated phalloidin (Molecular Probes) diluted 1∶100 in Alexa Fluor 568 anti-mouse antibody solution (Molecular Probes). Epifluorescence images were captured using an Olympus BX-51 fluorescence/DIC microscope with U plan Apo 40×/0.85 and 20×/0.70 objectives and an Olympus DP25 5.5 megapixel digital camera. Confocal images (0.2–0.3 *µ*m intervals in the Z-plane) were captured using a Zeiss LSM 510 META confocal microscope with F Fluar 40×/1.3 oil and Plan-Apo chromat 63×/1.3 oil objectives.

### Subcellular and Actin Fractionation

Cells were grown to visual confluence and harvested and scraped in 10 mM HEPES (pH 7.4), 1 mM EDTA, 0.25 M sucrose with protease inhibitor cocktail (Roche, Nutley NJ) and PhosStop phosphatase inhibitor cocktail (Roche). Cells were dounce-homogenized thirty times, and homogenates were spun at 2,500×g for 15 min to separate the nuclear pellet (P1) and cytosolic supernatant (S1). The S1 fraction was centrifuged in a TLA-100.3 rotor at 44,000 rpm at 4°C for 2 h to separate into S2 and P2 fractions. Fractions were resolved by electrophoresis and immunoblotted for pSYT, β1 integrin (1∶300; Santa Cruz), lamin-A/C (1∶200; Abcam), early endosome antigen-1 (EEA-1; 1∶1000; Pharmagen BD), and RhoGDI (1∶1000, Santa Cruz). The F- and G-actin pools were isolated using a Cytoskeleton Isolation Kit (BK039, Cytoskeleton, Denver CO). In brief, cell lysates treated with or without 4 µM of cytochalasin D were centrifuged at 50,000×g for 1 h to separate the G-actin (supernatant) and F-actin pools (pellet). The pellet was treated with 10% trichloroacetic acid and resuspended in 1/10 vol (v/v) relative to the G-actin supernatant. Equal aliquots of each were processed for immunoblotting.

### RNAi Ablation

Several RNAi duplexes, targeting both SYT mRNAs or only SYT/L mRNA, using BLOCK-iT^TM^ RNAi designer software (rnaidesigner.invitrogen.com). Sequences for RNAi duplexes are shown in Figure S6; the numbers used to designate each RNAi refers to the first base targeted (relative to the SYT/L mRNA). Stealth™ RNAi Negative Control (medium GC content; product number 12935–300; Invitrogen) duplexes were used as controls. U2OS or Cos-1 cells (1.2×10^6^) were plated on 6-well plates 1 d before transfection with Lipofectamine™ 2000 or Lipofectamine™ RNAiMAX (Invitrogen). Cells were then collected at 1–4 days or passed at 3 days post-transfection for adhesion assays. To generate pCMV-GFP SYT-NLS, we inserted full-length SYT/S cDNA the into the *Not*I site of pShooter™ (Invitrogen). To assess mRNA levels, cDNA was generated with a High-Capacity cDNA Archive kit (Applied Biosystems, Foster City CA) and amplified using primers that spanned exons 4–11, thereby amplifying both SYT/S and SYT/L transcripts. Sample loading and PCR efficiency were assessed by amplification of GAPDH cDNA.

### Adhesion and Spreading

U2OS cells (1.8×10^6^ viable cells) were plated on 10-cm culture dishes and, transfected with RNAi duplexes, and the numbers of attached and floating cells were determined daily for 3 days. Adherent cells were detached with 5 mM EDTA-PBS and suspended in serum-free McCoy5A medium, and 2–3.15×10^5^ viable cells were plated on specific ECM ligand precoated 6-well plates (BD Biosciences). After a 90-min incubation, wells were washed gently with PBS to remove unattached cells, and adherent cells were detached with EDTA-PBS. Cell counts were done with a hemocytometer. For spreading assays, U2OS cells were detached 3 days post-transfection with EDTA-PBS, and 3–4×10^5^ viable cells were plated on glass slides precoated with fibronectin or type I collagen (BD Falcon™ 8-well Culture Slides) or on chamber slides coated with 50 µg/ml ultra-pure laminin-111 or 100 µg/ml type IV collagen (BD Biosciences), as done in other studies [23], [42]. At 90 min or 5 h post-plating, slides were processed for immunostaining. Epifluorescent images were captured, and cell spreading was quantified using NIH Image J software as average area per cell.

## Supporting Information

Figure S1 RNAi Duplexes. (A) Sequences and Target Regions of SYT RNAi Duplexes. Shown are the RNAi duplexes used in these studies. The numbers indicate the position of the first nucleotide. (B-D). SYT mRNA and Protein Levels. U2OS cells were transfected with different RNAi duplexes, and the levels of SYT proteins were assessed 3 days later by immunoblotting and immunofluorescence with pSYT antibody and mRNA by RT-PCR. Bar = 20 µm.
(1.15 MB TIF)

Figure S2 Protein Sequencing. The identity the co-immunoprecipitated bands seen in Figure 3A,B was determined by tandem mass spectrometry (MS/MS). Bands were excised from SYPRO ruby-stained gels and sent to Midwest Bio services (http://www.midwestbioservices.com/index.html). At Midwest Bio, samples were trypsin digested, concentrated on a peptide trap column, and washed and desalted. The peptides were separated via microcapillary reverse-phase chromatography and sprayed directly into the mass spectrometer. Full MS and MS/MS spectra were acquired by an LCQ Deca XP Plus ion trap mass spectrometer (ThermoFinnigan). The sequences of the parent peptides were inferred by matching the MS/MS spectra to protein sequence databases using TURBOSEQUEST software. The peptides identified in the three excised bands are shown above. The co-immunoprecipitated bands were free of other products.
(0.12 MB TIF)

Figure S3 SYT does not Associate with Microtubules. (A,B) U2OS cells were exposed to 3 µg/ml nocodazole for 30 min. Whereas the microtubules were effectively disassembled, the filamentous strands of SYT remained intact. Bar = 10 µm (all panels). (C) NIH3T3 cells were immunostained with pSYT antibody and anti-tubulin antibody. Immunofluorescence signal for SYT did not colocalize with that for microtubules. This cell shows an absence of signal for nuclear SYT (dashed circle outlines the nucleus). We have found that in many cell types, the levels of nuclear SYT drop markedly during cytokinesis and recover hours later.
(4.62 MB TIF)

Figure S4 SYT is Required for Cell Spreading on Fibronectin and Laminin 111 but Not on Collagen. This experiment is a repeat of that shown in Figure 10, except that the cells were stained with rhodamine-conjugated phalloidin and DAPI and the images were captured at a lower magnification. U2OS cells were transfected with scrambled, panSYT229, panSYT471,or SYT/L RNAi duplexes. Three days later, the transfected cells were harvested and replated on chamber slides precoated with (A) fibronectin, (B) laminin-111, (C) type I collagen, or (D) type IV collagen. After a 90-min or 5-h incubation, the slides were processed for fluorescence staining. Bar = 40 µm. Whereas all cells with knock-down of total SYT (panSYT229 and panSYT471) show persistent impairment of cell spreading and formation of stress fibers and focal adhesions on fibronectin or laminin-111, a few cells with knock-down of SYT/L have begun to spread and form stress fibers at 5 h post-plating. In cells transfected with panSYT471 and SYT/L-specific RNAi duplexes spread and form stress fibers on either collagen type, very similar to control cells (scrambled RNAi). Cells transfected with RNAi panSYT229 do spread and form stress fibers on the collagen matrices.
(4.03 MB TIF)

Figure S5 Ablation of Total SYT Inhibits Cell Spreading and Stress Fiber Formation. U2OS cells were transfected with control or panSYT471 RNAi duplexes, transferred 2 days later to glass slides precoated with fibronectin or type I collagen. The cells were stained 24 h post-plating with rhodamine-conjugated phalloidin and antibodies against paxillin or pan-phosphotyrosine (P-Y). On fibronectin, adherent RNAi knock-down cells were unable to spread or form focal adhesions. In contrast, knock-down of total SYT with panSYT471 RNAi did not affect cell spreading or adhesion on type I collagen. Bar = 20 µm.
(4.32 MB TIF)

Figure S6 PCR Primers. Full length SYT cDNAs were cloned by RT-PCR of RNA from 293 cells using AccuPrime Taq (Invitrogen). PCR products were subcloned into pCMV-GFP to generate pCMV-GFP-SYT/S and pCMV-GFP-SYT/L. To generate an antigen common to both isoform, we amplified the 5̀ half of SYT cDNA upstream of the alternatively spliced exon 8 from pCMV-GFP-SYT/L. The PCR product was subcloned into the SmaI-HindIII site of pGEX-KG plasmid. For semi-quantitative RT-PCR, cDNA was synthesized from 3 µg of total RNA with a High-Capacity cDNA Archive kit (ABI). SYT cDNAs were amplified using primers that spanned exons 4-11, thereby amplifying both SYT/S and SYT/L transcripts.
(0.40 MB TIF)
