# Supplementary figures and images for: Cytosolic SYT/SS18 Isoforms Are Actin-Associated Proteins that Function in Matrix-Specific Adhesion
Source: PLoS One. 2009 Jul 31;4(7):e6455. doi: 10.1371/journal.pone.0006455 (PMC2714072; doi:10.1371/journal.pone.0006455)

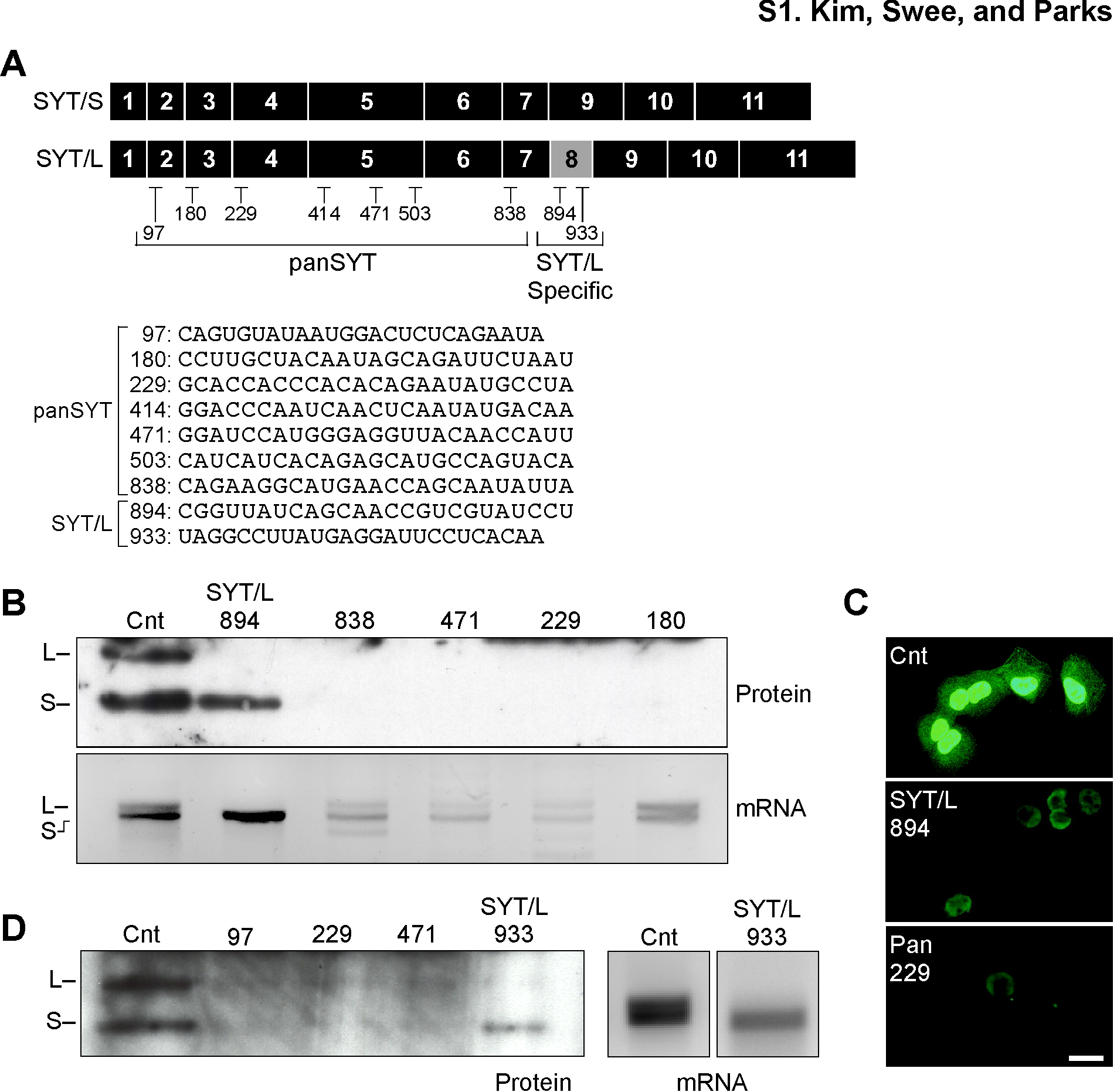

Supplement: Figure S1 — RNAi Duplexes. (A) Sequences and Target Regions of SYT RNAi Duplexes. Shown are the RNAi duplexes used in these studies. The numbers indicate the position of the first nucleotide. (B-D). SYT mRNA and Protein Levels. U2OS cells were transfected with different RNAi duplexes, and the levels of SYT proteins were assessed 3 days later by immunoblotting and immunofluorescence with pSYT antibody and mRNA by RT-PCR. Bar = 20 µm. (1.15 MB TIF) [file pone.0006455.s001.tif]

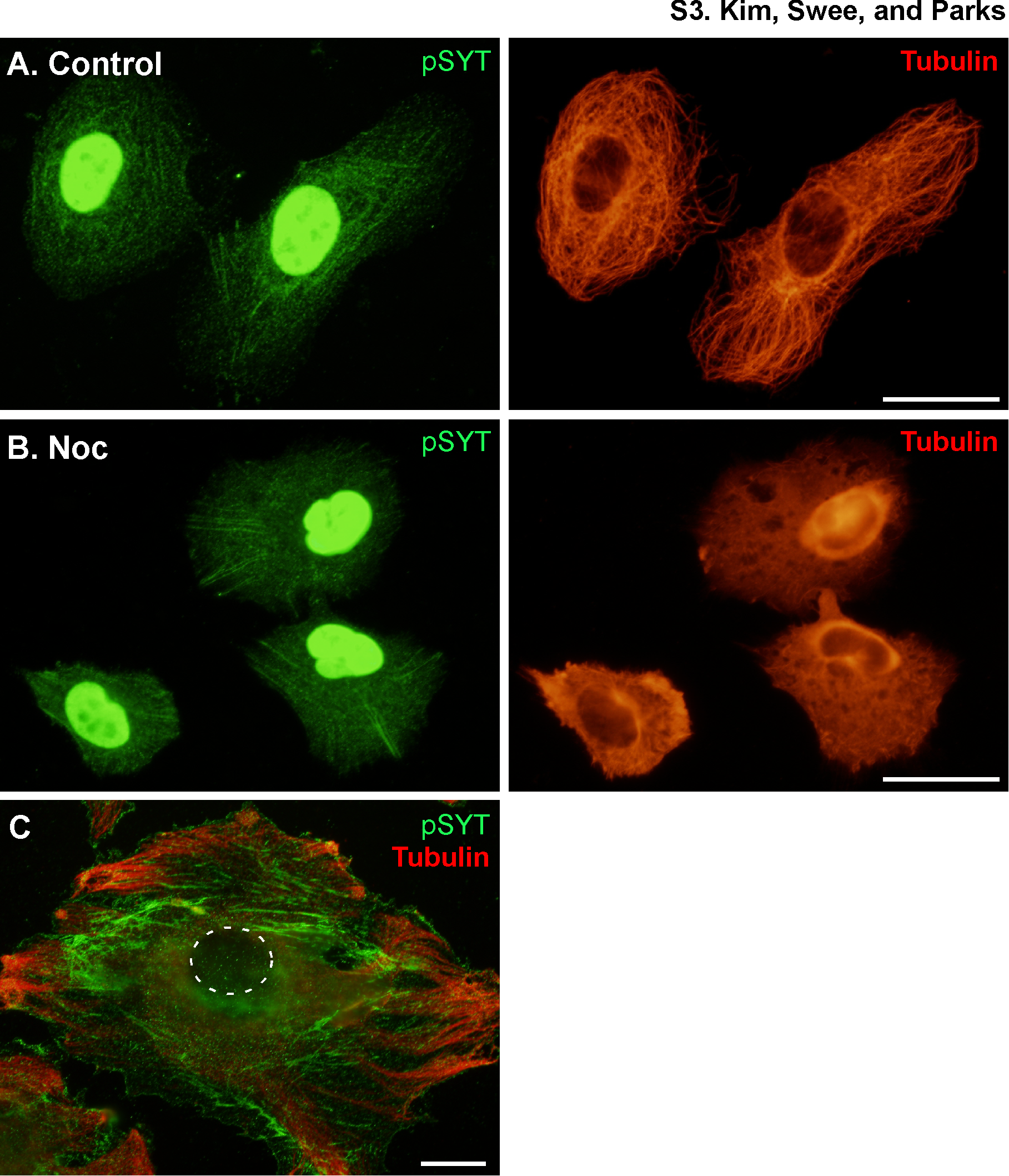

Supplement: Figure S3 — SYT does not Associate with Microtubules. (A,B) U2OS cells were exposed to 3 µg/ml nocodazole for 30 min. Whereas the microtubules were effectively disassembled, the filamentous strands of SYT remained intact. Bar = 10 µm (all panels). (C) NIH3T3 cells were immunostained with pSYT antibody and anti-tubulin antibody. Immunofluorescence signal for SYT did not colocalize with that for microtubules. This cell shows an absence of signal for nuclear SYT (dashed circle outlines the nucleus). We have found that in many cell types, the levels of nuclear SYT drop markedly during cytokinesis and recover hours later. (4.62 MB TIF) [file pone.0006455.s003.tif]

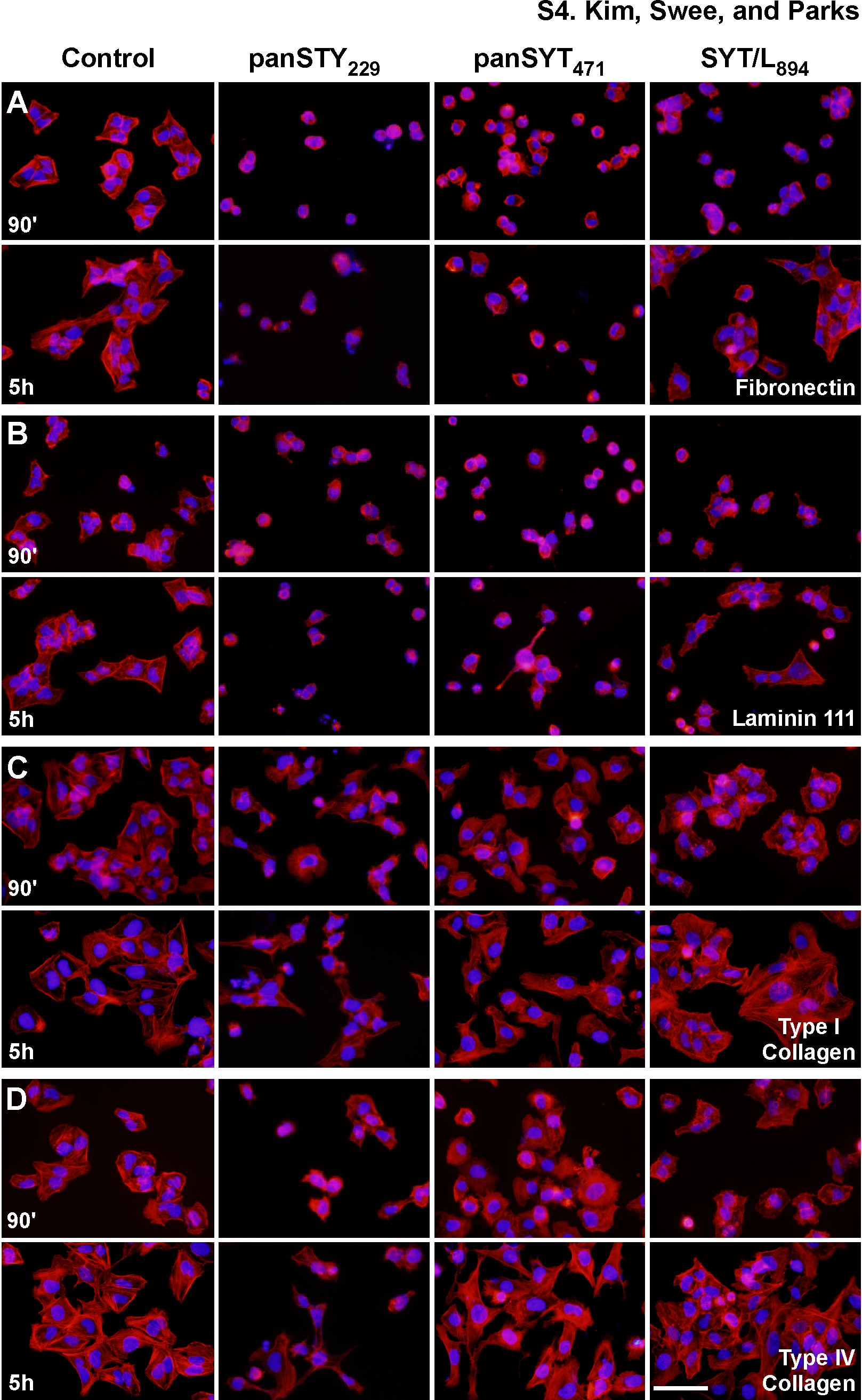

Supplement: Figure S4 — SYT is Required for Cell Spreading on Fibronectin and Laminin 111 but Not on Collagen. This experiment is a repeat of that shown in Figure 10, except that the cells were stained with rhodamine-conjugated phalloidin and DAPI and the images were captured at a lower magnification. U2OS cells were transfected with scrambled, panSYT229, panSYT471,or SYT/L RNAi duplexes. Three days later, the transfected cells were harvested and replated on chamber slides precoated with (A) fibronectin, (B) laminin-111, (C) type I collagen, or (D) type IV collagen. After a 90-min or 5-h incubation, the slides were processed for fluorescence staining. Bar = 40 µm. Whereas all cells with knock-down of total SYT (panSYT229 and panSYT471) show persistent impairment of cell spreading and formation of stress fibers and focal adhesions on fibronectin or laminin-111, a few cells with knock-down of SYT/L have begun to spread and form stress fibers at 5 h post-plating. In cells transfected with panSYT471 and SYT/L-specific RNAi duplexes spread and form stress fibers on either collagen type, very similar to control cells (scrambled RNAi). Cells transfected with RNAi panSYT229 do spread and form stress fibers on the collagen matrices. (4.03 MB TIF) [file pone.0006455.s004.tif]

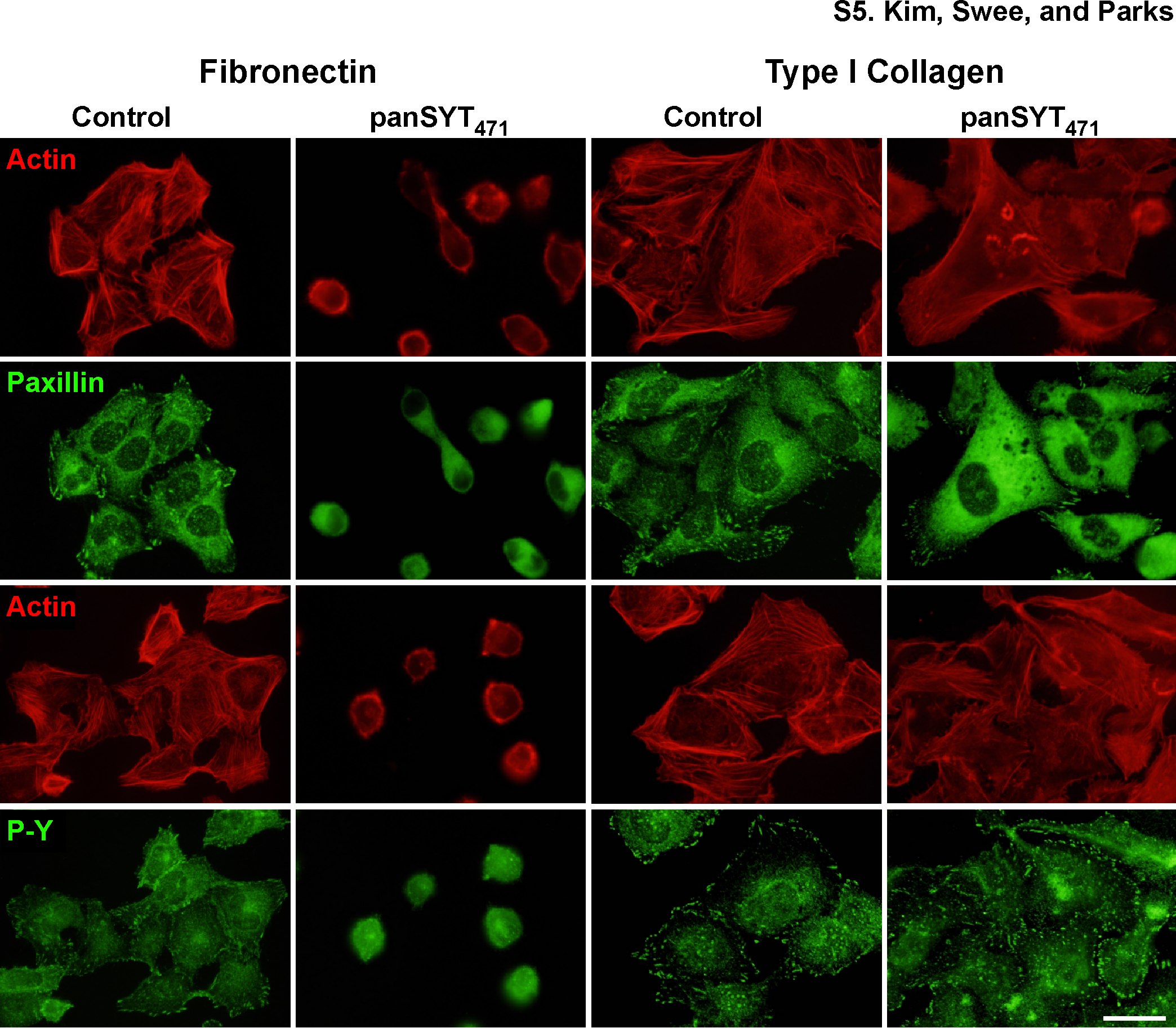

Supplement: Figure S5 — Ablation of Total SYT Inhibits Cell Spreading and Stress Fiber Formation. U2OS cells were transfected with control or panSYT471 RNAi duplexes, transferred 2 days later to glass slides precoated with fibronectin or type I collagen. The cells were stained 24 h post-plating with rhodamine-conjugated phalloidin and antibodies against paxillin or pan-phosphotyrosine (P-Y). On fibronectin, adherent RNAi knock-down cells were unable to spread or form focal adhesions. In contrast, knock-down of total SYT with panSYT471 RNAi did not affect cell spreading or adhesion on type I collagen. Bar = 20 µm. (4.32 MB TIF) [file pone.0006455.s005.tif]
